# Patient experiences of perioperative nutrition within an Enhanced Recovery After Surgery programme for colorectal surgery: a qualitative study

**DOI:** 10.1111/codi.13245

**Published:** 2016-02-02

**Authors:** V. Short, C. Atkinson, A. R. Ness, S. Thomas, S. Burden, E. Sutton

**Affiliations:** ^1^ NIHR Biomedical Research Unit in Nutrition, Diet and LifestyleUniversity Hospitals Bristol Education CentreBristolUK; ^2^ Head and Neck SurgeryUniversity Hospitals Bristol NHS TrustBristolUK; ^3^ School of Nursing, Midwifery and Social WorkUniversity of ManchesterManchesterUK

**Keywords:** Qualitative research, Enhanced Recovery After Surgery, colorectal surgery, patient experiences

## Abstract

**Aim:**

Nutrition is an important element of the Enhanced Recovery After Surgery (ERAS) programme. Patients have previously indicated that nutrition is a key component of ERAS that requires improvement. Our aim was to explore the perioperative nutrition experiences of colorectal surgical patients to identify barriers and facilitators to the integration of nutrition within ERAS.

**Method:**

Sixteen individuals undergoing colorectal surgery participated in a semi‐structured interview between postoperative day three and hospital discharge. The topic guide was developed iteratively throughout the study; topics included preoperative counselling, carbohydrate loading, fasting and postoperative nutrition. A constant comparison technique was employed during coding, and an inductive thematic analysis was used. Validity was ensured by double coding a sample of transcripts.

**Results:**

Findings are presented in the context of the following clinical themes: preoperative information, preoperative fasting, carbohydrate loading and nutritional drinks, postoperative diet and discharge. Individuals received too much general information which was repetitive, contradictory and not disease specific; this formed a key barrier affecting nutrition. Other barriers were negative experiences of nutritional drinks, stoma management, nausea and vomiting, and challenges from the hospital environment. Facilitators included interactions with staff, food accessibility and choice, and motivation for discharge.

**Conclusion:**

The key barrier to adherence of perioperative nutrition protocols was poor provision of information. Targeted information regarding postoperative diet, stoma management and coping with nausea and vomiting would be beneficial for colorectal surgical patients. Easily accessible food provided by ward staff was considered a facilitator.

**What does this paper add to the literature?**

Patient experiences of perioperative nutrition within Enhanced Recovery After Surgery (ERAS) have not previously been explored. This study used qualitative methods to provide a rich understanding of patient‐perceived barriers and facilitators to the uptake of ERAS nutrition components. Poor information was a key barrier; accessible staff support and motivation for discharge were important facilitators.

## Introduction

The Enhanced Recovery After Surgery (ERAS) programme is a multimodal perioperative care pathway 1 that improves clinical outcomes across surgical disciplines compared with standard care 2. For example, in colorectal surgery (CRS) reductions of 2.5–3 days in the length of hospital stay and up to a 50% decrease in the risk of complications have been observed, without evidence of increased readmissions 3, 4. Despite the evidence for the clinical benefit of ERAS, uptake and implementation have been slow 5 and audits and retrospective reviews indicate poor compliance with the protocol 6, 7. To improve compliance, it is important to explore experiences of ERAS to understand the barriers and facilitators affecting uptake of the programme. In ERAS, patients are encouraged to be active and responsible partners in the recovery process 8, therefore their views are important in assessing the uptake and implementation of ERAS pathways 9.

Although quantitative studies have investigated clinical benefits of ERAS, comparatively fewer studies have explored patients’ experiences, particularly using a qualitative approach 10. Qualitative methods can help evaluate patients’ experiences by identifying issues that would not arise within the prespecified boundaries of quantitative research 11. It has been recommended that complex interventions such as ERAS programmes should be assessed using both qualitative and quantitative methods 12, and this approach has previously been used successfully to explore patients’ experiences of various clinical programmes 13, 14.

Studies with a qualitative component investigating patients’ experiences of ERAS programmes have been undertaken in CRS 11, 15, 16, 17, 18, 19, gynaecological surgery 20, 21, 22 and liver surgery patients 23. None have focused specifically on the nutritional aspects of ERAS, despite studies suggesting that their uptake may independently predict reduced length of hospital stay and risk of complications 24, 25, 26. CRS patients in one study stated that it is the nutritional components of ERAS that most required improvement 16. This opinion has been reflected in surveys in which the nutritional ERAS items received the least positive ratings or the greatest variation in responses 9, 19, 27.

Our aim was to explore CRS patients’ experiences of perioperative nutrition within an ERAS programme in order to identify potential barriers and facilitators to the delivery and uptake of nutritional practices to inform future ERAS developments.

## Method

### Participants

Individuals scheduled for elective colorectal resection between October 2013 and March 2014 at the Bristol Royal Infirmary, Bristol, UK were recruited. Exclusion criteria were emergency surgery, age < 18 years, pregnancy, Crohn's disease, palliative surgery, poor spoken English, mental incapacity and individuals requiring barrier nursing. All participants were routinely entered onto an ERAS programme.

Purposive sampling was used to ensure approximately equal gender representation. Individuals were recruited until saturation (when no new themes emerged) 28. Thirty‐two individuals were approached. Sixteen consented and were interviewed. Reasons for noninclusion were unavailability of a researcher (*n *=* *1), discharge before the interview was scheduled (*n *=* *7), refusal to participate (*n *=* *3), transfer to intensive care (*n *=* *1) or surgery cancelled/delayed (*n *=* *4).

Approval was obtained from the NRES Committee East of England (Hatfield, reference 13/EE/0355) and the University Hospitals Bristol NHS Foundation Trust Research and Innovation Office. Participants gave written informed consent prior to participation, and study data were anonymized.

### Data collection

Participants took part in one semi‐structured bedside interview between postoperative day three and discharge. Interviews lasted approximately 40 min and were recorded using an encrypted audio recorder. Semi‐structured interviews permit discussion of key topics via a loose structure, whilst allowing respondents to raise further ideas 29. The initial topic guide was based upon ward meal time observations and previous literature, and it was developed iteratively throughout the study to explore emerging areas of interest 30 (Appendix S1). Topics included information provision, preoperative fasting, carbohydrate loading and postoperative diet. The topic guide, study documents and recruitment process were reviewed by a patient and public involvement (PPI) group.

The first four interviews were used as a pilot test of recruitment, the topic guide and the feasibility of ward interviews. The topic guide did not change substantially, hence these pilot interviews were included in the main dataset.

### Analyses

Audio recordings were transcribed verbatim and analysed using an inductive thematic analysis 31. Data were managed using nvivo 10 for Windows (v.10, 2010; QSR International Pty Ltd, Victoria, Australia). All transcripts were coded twice. The coding scheme was refined using the constant comparison technique to systematically address emerging concepts 32. Four transcripts were double coded by another qualitative researcher and coding schemes were compared to ensure reliability 33. Themes were identified from the codes and data they represented.

## Results

Sixteen participants were interviewed (nine men and seven women, aged 32–83 years; Table 1).

**Table 1:** Participant characteristics

Characteristics	Number of participants (*n*)
Age (years)^a^	68 (32–83) years
Gender (*n*):	
Males	9
Females	7
Ethnicity	Caucasian
Indication for surgery (*n*):	
Colorectal cancer	15
Metastases for appendiceal adenocarcinoma	1
Scheduled surgery type (*n*):	
High anterior resection	10
Low anterior resection	1
Right hemicolectomy	4^b^
Extra levator abdomino‐perineal excision	1
Stoma placement	8
Length of hospital stay (days)^a^	6 (4–21)
Postoperative complications (*n*)	5^c^

a Median (range).

b Right hemicolectomy aborted for two participants due to intraoperative complications.

c Abdominal distension, non‐functioning stoma, vasovagal episode, wound infection and ileus.

The thematic analysis results were grouped into the following clinically relevant themes: preoperative information, preoperative fasting, carbohydrate loading and nutritional drinks, postoperative diet and discharge.

### Preoperative information

Many participants were content with the general preoperative information provided, regarding this a means of emotional preparation for their forthcoming surgery. Some, however, expressed dissatisfaction at the lack of dietary information and were uncertain about who should provide this. Participants wanted a reliable source of nutritional information. The internet was viewed with mistrust and not considered dependable.

*… I've asked both the oncologist and the surgeon whether I should or should not be having or following a particular diet or avoid eating certain things and there was no specific information …* [participant 6, a 73‐year‐old man]

*I tried not to use the internet … because it would only frighten me …. I think more information about food would be good … what we should and shouldn't be eating …* [participant 13, a 60‐year‐old woman]

Many participants were older adults who found the volume of paper information hard to handle. Participants had difficulty comprehending the amount of information, indicating that it was not always relevant or consistent and therefore failed to meet their needs.

*… that thick the paperwork I went away from the, erm, the preop session with. It's a good job I've got a blue pass because the car was right under the hospital, else I simply couldn't have got it there*. [participant 9, a 71‐year‐old man]

*… it was all repetitious, to me … half of books were waste of time to what I was having done … there was twice I rang them up. But that was because they give me contradictory things*. [participant 8, a 66‐year‐old man]

### Preoperative fasting

Most participants understood the fasting requirements and did not report difficulty fasting preoperatively. Some reported conflicting advice about preoperative fasting in the leaflets provided compared with verbal face‐to‐face information given by the specialist nurse. Where this occurred individuals followed the nurse's advice upon clarification.

*It* [preoperative fasting period] *was no problem at all*. [participant 15, a 67‐year‐old man]

*… that* [leaflet] *says you can eat up to something like 6 pm on the day before the op. But I remembered [nurse], the nurse here, saying I couldn't have anything the entire day before …. I was able to ring up and check that that was the case …. I thought maybe I'd misheard … what* [nurse] *had said*. [participant 16, a 65‐year‐old‐man]

### Carbohydrate loading and nutritional drinks

Most participants were aware of when they were expected to consume drinks preoperatively, but were unclear on differences between carbohydrate loading and nutritional supplement drinks. Participants did not find the drinks pleasant, but viewed them as important to ‘build up’ and ‘prepare’ for surgery. However, participants were less willing to consume nutritional drinks postoperatively.

*… it was, “What do you want for a drink?” and the first couple of days* [postoperatively] *I said, “Yes” and then I thought, “I don't really enjoy these, they are just so sweet”. So I didn't bother*… [participant 7, a 73‐year‐old man]

### Postoperative diet

Many participants were aware of the guidance to eat normally as soon as possible, and several individuals tolerated food when it was first offered postoperatively. Individuals generally were content with the notion of rapid resumption of diet and were pleased to be responsible for this aspect of their recovery. However, some reported reduced appetite due to vomiting or intense nausea, or the fear of these occurring. This limited participants’ willingness to resume diet, as individuals were distressed at the thought of vomiting again or wanted to rest the digestive system. Some participants also considered that reduced appetite and food intake were understandable whilst in hospital, due to their lower activity levels compared with when they were at home.

*… when I've been sick, I feel I want to give my stomach a rest … once I've been sick I will tend to say … “Well, I won't have any more food now for a while”*. [participant 10, a 73‐year‐old man]

Additionally, placement of a stoma caused difficulties for some participants. Individuals found it challenging to accept such an extreme bodily alteration, particularly as this appeared to cause substantial changes in their perception of food: food was now viewed as a burden. Participants found the idea of adjusting their normal activity and food intake to accommodate the stoma challenging. Individuals were unclear which foods would be best tolerated and which might aggravate stoma output or cause blockage. One individual reported attempts to reduce food intake in an effort to decrease how frequently the stoma bag would require emptying.

*… everything that goes in my mouth I can see coming out and that's quite off‐putting …. I think subconsciously I'm probably holding back more on what I'm eating because I know ultimately in a few hours I'm gonna have to open that up and get rid of it …* [participant 3, a 32‐year‐old woman]

Staff were considered helpful in assisting individuals and providing access to food. They were perceived as approachable; consequently, individuals felt confident in asking for food and advice. Participants were grateful for the staff's caring attitude, and felt reassured that they were receiving high quality service.

*You can always ask them for a sandwich … whatever day, time of the day and night it is and they will get it for you*. [participant 8, a 66‐year‐old man]

Participants were also generally impressed with the choice of food available, although there was some concern that the choice compromised the food quality.

*… there are far too many things on the menu … they should do a few things much better …* [participant 6, a 73‐year‐old man]

### Discharge

Participants were keen to be discharged as early as possible. Returning home was considered an important step in recovery, and individuals understood that resuming diet was a discharge criterion. Despite lack of appetite and challenges such as nausea and vomiting, the prospect of returning home acted as a strong motivator for participants to begin eating postoperatively.

*I'm gonna try a bit again today … obviously I've got to eat my food before I can go home … if I don't eat then I won't be allowed to go home …* [participant 12, a 58‐year‐old woman]

Participants considered that going home would also enable return of their appetite to its original ‘normal’ state. Individuals were keen to pursue a sense of normality, and therefore were eager to engage in recovery activities to promote an earlier return home. The concept of normality associated with discharge was perceived as a facilitator for postoperative food consumption. However, some individuals were concerned about possible lifestyle changes when returning home (slowing down activity, altering diet), and were apprehensive about establishing a new sense of normality.

*… I was told … when I go home and I'm able to eat again, I can eat or drink anything I want … hopefully I'll go back to how I was eating and drinking before*. [participant 11, a 69‐year‐old woman]

## Discussion

Our study identified several barriers and facilitators to meeting the ERAS recommendations for perioperative nutrition. Patient information was of particular concern. There was too much of it and it was repetitive and sometimes contradictory. There was also a lack of detail about nutrition with a stoma and when feeling nauseous. Additional barriers included the unpleasant taste of nutritional drinks and food quality. Participants responded positively to the variety and accessibility of food. The desire to return home also motivated individuals to begin eating.

Previous studies have not specifically explored experiences of the nutritional components of ERAS. Key topics identified in our study show similarities with previous interviews, focus groups and questionnaires considering colorectal and gynaecological patients' experiences of ERAS. These include the importance of information provision, control and involvement in recovery, anxiety about the nutritional supplement drinks, nausea, concern regarding the quality and unappetising nature of hospital food and the desire to go home 15, 16, 17, 18, 19, 20, 21, 22.

Information provision allows patients to feel prepared, play an active role during recovery and understand their care process 16, 20, 34. Interviews with CRS patients in Sweden showed that the provision of information is important for building trust between patients and care providers 34. Patients’ desire to feel in control (facilitated by information provision) was also observed, a concept witnessed in our study. Previous studies using questionnaires, interviews and focus groups with people undergoing CRS and gynaecological surgery have also indicated that patients feel overloaded with general information but lack specific dietary advice 9, 16, 27, 34. Participants in our study reported similar views, requesting more nutritional information.

Similar to the findings of Taylor and Burch 16, participants in the present study did not enjoy the nutritional supplement drinks. Individuals tolerated these preoperatively as a form of preparation for surgery but were reluctant to continue postoperatively. For some participants this was due to nausea or reduced appetite. Another potential reason alluded to during interviews, but not stated specifically, was that the supplement drinks had been consumed when participants felt they were most needed (i.e. preoperatively) and that further compliance (i.e. postoperatively) was less important. Individuals in the study discussed how vomiting (or fear of it) hindered food intake, similar to reports from focus groups with CRS patients 16. Conversely, a recent interview study indicated that symptoms such as nausea did not deter patients from active engagement in recovery 18. In our study, placement of a stoma caused anxiety regarding food consumption for some individuals. The influence of a stoma has not been explored in this context in previous qualitative studies of ERAS experiences.

A range of views were expressed in the present study during discussions about hospital food. This parallels findings from two questionnaire‐based studies (in the UK and Australia) in CRS patients in which questions about hospital food showed the greatest variation in responses compared to other ERAS components 9, and where food quality received the poorest responses 27. Research in Australia, Switzerland and the UK has indicated that choice of food is important for patient satisfaction 35, 36, 37, 38. Participants in our study agreed that the choice of food was sufficient and that food was easily accessible via staff, a view shared by individuals in another study 36. Conversely, a previous study identified that access to food was considered problematic for patients 39. This may reflect recent improvements in hospital food provision and accessibility, or simply differences in food provision between hospitals.

Participants in our study associated being at home with recovery and restoration of normality. This perception has previously been observed from interviews with people undergoing CRS 11. A recent study also found that going home was considered a motivator driving active recovery, although once home individuals were worried about re‐establishing normality in daily life with the added burden of changes in bowel function and pain 18.

The present study suggests that patients would like more detailed nutritional information, with clear, consistent and concise messages. The availability of a trusted and easily accessible source of information was considered important. Participants prioritized verbal face‐to‐face information from the specialist nurse over pre‐prepared leaflets, potentially due to greater trust and the belief that such information is individualized. Information in this format may be easier to comprehend, suggesting a greater effectiveness of information transmitted verbally. Given the time constraints faced by staff an alternative approach might be a DVD, although the suitability of this may be limited by access to appropriate technology. To aid greater postoperative acceptance of nutritional drinks, hospitals could ensure the availability of and access to a variety of flavours. In addition, more advice and reassurance regarding handling a stoma (in respect to diet) and nausea could aid individuals. In our hospital, the current availability and choice of hospital food should be maintained but ideally higher quality food provided. This may, however, have cost implications. Individuals felt confident in accessing food via staff, demonstrating the importance of interaction with staff to enable an early resumption of diet.

A key strength of our study is the focus on nutrition. To our knowledge, this is the first study to investigate patients’ experiences of perioperative nutrition within an ERAS context. Another strength is the study design and method: purposive sampling ensured approximately equal representation across genders, semi‐structured interviews enabled similar discussion topics across participants, while allowing respondents to elaborate freely, iterative development of the topic guide permitted participants to discuss factors raised in earlier interviews, the constant comparison technique enabled refinement of the coding scheme as data were collected and double coding four transcripts ensured reliability of the coding scheme.

Although our study focused on one surgical discipline within one hospital, this permitted an in depth exploration of a specific group of patients. The findings may be applicable to other populations as ERAS spreads across surgical disciplines. We included a relatively small sample size, but recruitment continued until saturation, and it is unlikely that a larger sample would have produced new themes. Interviews were conducted at participants’ bedsides, thus individuals may have altered responses if staff were present nearby. However, in agreement with previous studies in nonclinical environments 16, 27, interactions with staff were extremely positive with little criticism, suggesting that interview location may have had minimal effect on study findings.

Future studies could explore the acceptability and effectiveness of different methods of providing information. Additionally, studies of postoperative changes in food preferences and appetite, as described by Welchman *et al*. 40, could aid provision of more palatable food to encourage postoperative intake. The available relevant qualitative literature has largely focused on CRS patients and exploration of experiences in other surgical populations may also be useful.

In summary, general information provided to patients during the perioperative period should be more concise, clearer and targeted. Furthermore, the availability and accessibility of information should be improved. Verbal face‐to‐face information is preferred, but cost implications may be a barrier to this. There is a need for more dietary advice, particularly in relation to a stoma, as stoma patients appeared to be especially vulnerable. Similarly, advice on how to manage nausea and vomiting would be welcomed and may enable patients to feel adequately prepared and in control of their recovery. In order to facilitate early resumption of an oral diet, patients should be made aware that food can be easily accessed at all times from ward staff, and staff should be encouraged to motivate patients to eat. The requirement to tolerate food before discharge should also be reiterated to patients.

## Conflicts of interest

The authors declare no conflict of interest. This work was supported by the NIHR Biomedical Research Unit in Nutrition, Diet and Lifestyle, University Hospitals Bristol. Vaneesha Short was supported by a University of Bristol PhD scholarship.

## Supporting information


**Appendix S1.** Topic guide.
