# Supplementary material for: Patient experiences of perioperative nutrition within an Enhanced Recovery After Surgery programme for colorectal surgery: a qualitative study
Source: Colorectal Dis. 2016 Feb 2;18(2):O74–80. doi: 10.1111/codi.13245 (PMC4755035; doi:10.1111/codi.13245)
Supplement: Supplementary file 1 — Appendix S1. Topic guide. [file CODI-18-O74-s001.docx]

| **Nutritional information**   1. If you can think back to before you came into hospital, were you provided any information about nutrition?  - advice for diet *before* coming in to hospital - advice on when to *stop* eating before surgery - advice on what to *expect* from the meal service in hospital - advice on when you should *start* eating after surgery - *when* this information was provided - was there the opportunity to *ask questions* - was there the opportunity to *seek advice* on several occasions  1. What nutritional information was provided?  - *what form this was in: leaflet/face to face explanation etc.* - *what the information consisted of*  1. Who provided the nutritional information? Did you seek any information yourself? [*Prompt: nurse, dietician etc.*] 2. How did you feel about the information provided?  - did you feel *prepared* for what to expect in hospital (in terms of the food) - was the information *relevant* to you - were you provided it *alone* or with a partner etc. - did you feel *reassured* about staying in hospital/food provided - were you told anything that you *weren’t expecting*  1. Did you make any lifestyle changes before surgery in response to the information given? [*Prompt: dietary, exercise, smoking etc.*]   **Preoperative Nutrition**   1. Were you offered any preoperative carbohydrate drinks? [*Prompt: if yes, Why/why not did you decide to take them?; if not*: *Would you have liked to have been offered them?*] 2. What was your understanding of why you were offered them?  - did you know what the reasons for drinking them were - did you understand at what time points to drink them - were you offered enough information about them  1. What did you think of them? [*Prompt: taste, aroma, volume, how long before surgery you drank them*] 2. When did you stop eating before surgery? What did you eat before surgery? | **Postoperative nutrition**   1. What were your expectations for the food and meals in hospital?  - *when* did you expect to start eating - *what* did you expect the food to be like - any *previous experience* of hospital food/experiences of others  1. What did you think of the food and meals provided?  - when did you *start* eating - what was the *first thing* you ate - what sort of *other food* have you been eating - what were you *offered* - did you feel *anxious* about eating after surgery - *nausea/vomiting* - what was the *service* like - were the *servers* helpful - what did you think of the presentation of the food, aroma, texture, choice, availability, accessibility, timing of meals - what did you *want* to eat  1. How did you feel you were expected to behave in terms of eating postoperatively?  - *when* did you feel you should *start eating* - *what* did you feel you should be eating - *how much* did you feel you should be eating - did you feel *ready* to start eating when you did - how did you feel about having *control of eating* postoperatively  1. Was there anything that you felt stopped you from starting to eat sooner? [*Prompt: hospital setting, staff, medications, mobility, pain etc.*] 2. Was there anything you felt helped you start eating quickly after surgery? [*Prompt: staff encouragement, food from home etc.*] 3. What do you think hospitals could do better to improve the nutritional experience of surgical patients? [*Prompt: food factors, staff, environment, timing etc*] |
| --- | --- |
|  | **General**  Is there anything that you would like to mention that you don’t think we’ve covered? |
